# Isolated choanal and gut atresias: pathogenetic role of serine protease inhibitor type 2 (*SPINT2*) gene mutations unlikely

**DOI:** 10.1186/s40001-018-0312-2

**Published:** 2018-03-02

**Authors:** Christian Niederwanger, Silvia Lechner, Lisa König, Andreas R. Janecke, Claus Pototschnig, Beatrice Häussler, Sabine Scholl-Bürgi, Thomas Müller, Peter Heinz-Erian

**Affiliations:** 1 Department of Pediatrics III, Medical University of Innsbruck, Anichstrasse 35, 6020 Innsbruck, Austria; 2 VAMED, 1230 Vienna, Austria; 3 Department of Pediatrics I, Medical University of Innsbruck, Anichstrasse 35, 6020 Innsbruck, Austria; 4 Department of Ear, Nose and Throat Diseases, Medical University of Innsbruck, Anichstrasse 35, 6020 Innsbruck, Austria; 5 Department of General Surgery, Pediatric Surgery Unit, Medical University of Innsbruck, Anichstrasse 35, 6020 Innsbruck, Austria

**Keywords:** Isolated atresia, Gut, Choanae, Serine protease inhibitor type 2, *SPINT2* splice mutation

## Abstract

**Background:**

Choanal (CA) and gastrointestinal atresias (GA) are an important feature of syndromic congenital sodium diarrhea (sCSD), a disorder recently associated with mutations in the gene for serine protease inhibitor type 2 (*SPINT2*). It is, however, not known whether isolated non-syndromic CA and GA themselves might result from *SPINT2* mutations.

**Methods:**

We performed a prospective cohort study to investigate 19 CA and/or GA patients without diarrhea (“non-sCSD”) for potential sCSD characteristic clinical features and *SPINT2* mutations.

**Results:**

We found a heterozygous *SPINT2* splice mutation (c.593-1G>A), previously demonstrated in sCSD in homozygous form, in only 1 of the 19 patients of the “non-sCSD” cohort. This patient presented with isolated anal atresia and borderline low laboratory parameters of sodium balance. In the remaining 18 non-sCSD CA/GA patients investigated, *SPINT2* sequence analysis and clinical markers of sodium homeostasis were normal. None of the 188 healthy controls tested in a regional Tyrolean population harbored the c.593-1G>A mutation, which is also not listed in the ExAc and gnomAD databases.

**Conclusions:**

The finding of only one heterozygous *SPINT2* mutation in 19 patients with isolated CA/GA was not statistically significant. Therefore, *SPINT2* mutations are an unlikely cause of non-sCSD atresia.

*Trial registration* ISRCTN73824458. Retrospectively registered 28 September 2014

## Background

Atresias of the choanae (CA) [1] and the gut (GA) have been shown to be due to a large variety of pathomechanisms [2–6]. One disorder also commonly exhibiting CA and/or GA is autosomal recessive syndromic congenital sodium diarrhea (sCSD) [7]. The phenotype of sCSD involves voluminous watery stools and enormous fecal losses of sodium, leading to hyponatremia and metabolic acidosis. The disease is caused by homozygous and compound heterozygous mutations in the *SPINT2* gene [7]. *SPINT2* encodes for Kunitz-type serine protease inhibitor type two, also reported as placental bikunin [8] and hepatocyte growth factor activator type 2 [9], which has been shown to inhibit a number of serine proteases such as matriptase, prostasin and furin [10–12]. The latter have been postulated to regulate the activity of the epithelial sodium channel (ENaC) which allows uptake of sodium by many cell types, including the intestinal epithelium [12]. *SPINT2* mutations in sCSD patients may interfere with the normal process of regulated proteolytic activity and thus lead to defective function of ENaC. This could result in enhanced diarrheal sodium loss via the gut epithelium [7] as well as frequent occurrence of atretic malformations in sCSD, although the pathomechanism is currently incompletely understood. We investigated patients with isolated forms of CA and GA, who did not suffer from sodium-losing diarrhea (non-sCSD atresia), with regard to *SPINT2* mutations and clinical picture, and compared the results with those from sCSD patients.

## Patients and methods

The study was approved by the ethics committee of the Medical University of Innsbruck. 84 patients diagnosed with CA and/or GA between January 1, 1980 and December 31, 2011 were identified from the patient registries of the University Hospitals of Innsbruck and invited for a clinical examination. 19 patients (9 males, 10 females, aged 2–40 years) were included in the study after giving written informed consent. A careful history and physical examination by an ear nose and throat (ENT) specialist, a pediatric surgeon and a pediatrician was performed. Type and localization of the respective atresia was determined from previous history and physical examination data, diagnostic imaging results and surgery reports. Laboratory workup consisted of electrolytes, blood gases, osmolality and creatinine in venous plasma as well as of electrolytes, osmolality and creatinine in urine. The concentrations of sodium and creatinine in plasma and urine were used for the calculation of the fractional excretion of sodium (FENa) using the formula FENa = urine Na × plasma creatinine × 100/(plasma Na × urine creatinine). Concentrations of uNa (urine sodium) and uCrea (urine creatinine) were used for the calculation of the urine sodium to urine creatinine (uNa/uCrea) ratio. Furthermore, stool samples were analyzed for electrolytes, pH and osmolality.

*SPINT2* mutational analysis was performed as described previously [7]: briefly, genomic DNA was prepared from leukocytes using a robot (GenoM 48, Qiagen, Vienna, Austria). The complete coding region of the *SPINT2* gene and all exon–intron-boundaries were amplified using intronic primers. The 190–504-bp amplicons were sequenced and the sequencing reactions analyzed on an ABI 3100 DNA sequencer. A panel of 188 DNA samples from anonymous healthy subjects was investigated for the presence of sequence variants [7]. Clinical and mutational analysis data of the 19 non-CSD atresia patients were compared with published results from 16 patients with sCSD [7]. Data of clinical parameters are presented as mean ± SD. The statistical significance of differences between both patient groups was determined using the two-tailed Student’s *t* test. Allele frequencies for *SPINT2* variants were taken from the ExAC and gnomAD databases.

## Results

Among non-sCSD CA or GA patients, no homozygous or compound heterozygous mutations were found in the present study (Table 1 upper panel). However, two heterozygous *SPINT2* nucleotide alterations were identified in exon seven of two non-sCSD patients with anal atresia. c.598G>C het, demonstrated in patient #10 (Table 1), was found to be a common polymorphism with an allele frequency of 2.8% in 61.000 individuals (ExAC database). c.593-1G>A het, detected in a second non-sCSD atresia patient (patient #14, Table 1 upper panel) of our study is a splice mutation that had been identified in homozygous state in five previously reported Austrian sCSD patients [7]. This mutation affects the canonical acceptor splice site in intron 6 of the *SPINT2* gene. It is not listed in public databases of population-based exome and genome data, i.e., neither in ExAC (http://exac.broadinstitute.org/gene/ENSG00000167642) nor in gnomAD (http://gnomad.broadinstitute.org/gene/ENSG00000167642). It emerged that all five homozygous patients and the heterozygous patient #14 of the present study originated from the same rural area, suggesting that patient #14 of the non-sCSD group most likely was a heterozygous sCSD carrier.

**Table 1:** Clinical and molecular data of non-CSD and sCSD patients

Patient #	Actual age (months)	Sex (m/f)	GA (months)	BW (g)	ppH	pNa (mmol/L) (136–145)	uNa (mmol/L) (30–250)	pCrea (mmol/L) (0.05–0.1)	uCrea (mmol/L) (3.5–24.6)	FENa (%) (0.5–1.5)	uNa/uCrea (mmol/mmol) (17–53)	fNa (mmol/L) (20–50)	fpH (6.5–7.5)
Non-sCSD													
1	180	M	39	3270	7.38	140	101	0.09	3.8	1.7	26.6	28	5.0
2	156	M	40	3360	7.37	140	129	0.10	7.0	1.3	18.4	17	7.5
3	84	M	37	2510	7.38	138	54	0.06	3.5	0.7	15.4	29	7.5
4	480	F	n.i.	2500	7.39	141	248	0.10	6.2	2.8	40.0	32	5.5
5	96	M	33	1835	7.29	139	192	0.05	9.2	0.8	20.9	38	8.0
6	108	F	38	3650	7.20	142	161	0.05	9.7	0.6	16.6	24	7.0
7	184	M	39	3350	7.14	145	199	0.06	9.2	0.8	21.6	24	5.5
8	158	M	39	2700	7.30	139	n.i.	0.05	n.i.	n.i.	n.i.	39	9.0
9	144	F	n.i.	3270	7.35	140	133	0.04	9.3	0.4	9.3	14	6.0
10	49	F	39	2760	7.36	138	100	0.06	4.0	1.1	25.0	30	5.5
11	192	F	39	3200	7.40	136	178	0.05	18.1	0.4	9.8	n.i.	6.5
12	60	F	41	4010	7.38	138	21	0.05	0.8	1.0	26.3	19	6.0
13	86	F	41	3880	7.36	139	183	0.06	4.5	1.8	40.7	n.i.	n.i.
14	146	M	40	3440	7.33	141	28	0.05	1.7	0.6	16.5	21	6.0
15	88	F	38	3185	7.36	143	143	0.05	3.0	1.7	47.7	31	5.5
16	252	F	41	3820	7.39	139	178	0.05	7.4	0.9	24.1	30	7.5
17	25	M	37	2510	7.38	140	106	0.05	2.4	1.0	27.6	30	6.5
18	85	F	40	3030	7.35	139	124	0.06	3.9	2.5	31.8	49	6.0
19	74	M	39	3305	7.37	141	225	0.05	5.7	1.4	39.5	37	6.0
sCSD													
1	13	M	35	2850	7.04	119	< 10	0.19	17.7	< 0.1	0.6	152	8.1
2	7	F	38	2910	7.11	123	< 10	0.16	14.2	< 0.1	0.7	190	8.6
3	6	M	32	2190	n.i.	n.i.	n.i.	n.i.	n.i.	n.i.	n.i.	n.i.	n.i.
4	10	F	36	2900	6.99	119	16	0.08	10.0	< 0.1	1.6	105	7.5
5	4	F	32	2190	7.13	118	< 10	0.11	9.0	< 0.1	1.1	103	7.8
6	98	F	37	2670	7.09	116	< 10	0.12	11.2	< 0.1	0.9	133	8.0
7	6	F	40	2995	7.33	120	n.i.	0.06	n.i	n.i.	n.i.	100	n.i.
8	14	F	39	3345	7.25	129	n.i.	0.07	n.i.	n.i.	n.i.	106	n.i.
9	192	M	40	3635	n.i.	119	n.i.	0.09	n.i.	n.i.	n.i.	90	6.0
10	180	F	39	3620	n.i.	119	n.i.	1.1	n.i.	n.i.	n.i.	86	5.5
11	180	F	35	2540	7.22	119	n.i.	0.09	n.i.	n.i.	n.i.	90	6.0
12	31	M	36	3130	7.27	123	n.i.	0.09	n.i.	n.i.	n.i.	56	7.0
13	11	F	40	3050	7.21	116	n.i.	0.08	n.i.	n.i.	n.i.	66	n.i.
14	22	F	36	3075	7.30	124	n.i.	0.08	n.i.	n.i.	n.i.	92	8.2
15	108	M	36	2400	7.19	125	3	0.15	6.0	< 0.1	0.5	95	n.i.
16	72	F	n.i.	n.i.	n.i.	n.i.	10	n.i.	7.0	n.i.	n.i.	119	n.i.

Patient #	Actual age (months)	Sex (m/f)	Diarrhea	Atresia	*SPINT2* nucleotide alteration	Diagnosis
Non-sCSD						
1	180	M	No	Choanal	None	CA
2	156	M	No	Choanal	None	CA
3	84	M	No	Esophageal	None	EA IIIb
4	480	F	No	Esophageal	None	EA
5	96	M	No	Esophageal	None	EA IIIb
6	108	F	No	Esophageal	None	EA IIIb
7	184	M	No	Esophageal	None	EA
8	158	M	No	Anal	None	AA
9	144	F	No	Anal	None	AA
10	49	F	No	Anal	c.598G>C het	AA
11	192	F	No	Anal	None	AA
12	60	F	No	Anal	None	AA
13	86	F	No	Anal	None	AA
14	146	M	No	Anal	c.593-1G>A het	AA
15	88	F	No	Anal	None	AA
16	252	F	No	Anal	None	AA
17	25	M	No	Anal	None	AA
18	85	F	No	Anal	None	AA
19	74	M	No	Anal	None	AA
sCSD						
1	13	M	Yes	Choanal, corneal erosions	c.593-1G>A homozygous	
2	7	F	Yes	Corneal erosions	c.593-1G>A homozygous	sCSD
3	6	M	Yes	n.i.	c.593-1G>A homozygous	
4	10	F	Yes	Corneal erosions	No DNA, sister of pat. 3	sCSD
5	4	F	Yes	Choanal	c.593-1G>A homozygous	sCSD
6	98	F	Yes	None	c.593-1G>A homozygous	sCSD, ON
7	6	F	Yes	Choanal	c.488A>G homozygous	sCSD
8	14	F	Yes	Choanal	c.488A>G homozygous	sCSD
9	192	M	Yes	None?	c.488A>G homozygous	sCSD, PPN
10	180	F	Yes	Choanal	c.488A>G homozygous	sCSD, PPN
11	180	F	Yes	Choanal	c.337 + 2T>C/c.488A>G	sCSD, PPN
12	31	M	yes	Choanal	c.488A>G homozygous	sCSD
13	11	F	Yes	Choanal, anal	No DNA, sister of pat. 12	sCSD
14	22	F	Yes	Choanal, corneal erosions	c.488A>G/c.553+2T>A	sCSD
15	108	M	Yes	Choanal	c.488 <A>G/c.1A>T	sCSD
16	72	F	Yes	Choanal, anal	c.488A>G homozygous	sCSD, PPN

Abbreviations in alphabetical order: *AA* anal atresia; *BW* birth weight; *CA* choanal atresia; *EA* esophageal atresia; *fNa* fecal Na; *fpH* fecal pH; *f* female; *FENa* fractional excretion of sodium; *GA* gestational age; *GIA* gastrointestinal atresia; *m* male; *mo* month; *n.a*. not applicable; *n.i*. no information; *ON* oral nutrition; *pNa* plasma Na; *ppH* plasma pH; *PPN* partial parenteral nutrition; *uNa* urinary Na; *uNa/uCrea* urinary Na:urinary creatinine ratio

The individual clinical data of the non-sCSD atresia and sCSD patients are summarized in Table 1. The average age of the non-sCSD atresia group was 139 ± 101 months (range 25–480 months) and thus significantly higher (*p* < 0.05) than the age of the sCSD patients (60 ± 70 months, range 4–192 months). The gestational age of non-sCSD atresia patients was also significantly higher than that of the sCSD group (38.8 ± 1.9 vs. 36.7 ± 2.6 weeks, *p* < 0.05). Birth weight which was 3136 ± 554 g for non-sCSD atresia patients and 2900 ± 448 g for the sCSD group was not significantly different (*p* = 0.19). Diarrhea was not reported in any of the non-CSD atresia subjects, while it was the predominant feature in all sCSD patients. Moreover, average values of laboratory indicators of sodium balance (fecal Na, plasma Na, urinary Na, FE_Na_ and uNa/uCrea ratio) and of metabolic acidosis were normal in the non-sCSD atresia group and were thus significantly different from the sCSD cohort where these parameters were generally pathological (Table 2). There was also no diarrhea reported in the non-sCSD atresia patient with the heterozygous splice mutation c.593-1G>A (patient #14). However, the FENa and uNa/uCrea in that patient showed borderline low values for these two sodium balance parameters and also a rather low uNa concentration (Table 1). We speculate that these results reflect an effect of the c.593-1G>A carrier state on sodium status, because we had previously found low urinary sodium concentrations in two obligate heterozygous sCSD parents [13]. This resembles findings in other autosomal recessive disorders, where clinically healthy patients have been shown to exhibit pathological laboratory markers as in, e.g., alpha-1-antitrypsin deficiency [14, 15]. Apart from patient #14, however, the results in all other 18 non-sCSD patients were normal. Besides the findings in the non-sCSD cohort, the absence of the c.593-1G>A mutation among 188 ethnically matched controls argued against a role for *SPINT2* in isolated CA/GA.

**Table 2:** Clinical parameters reflecting differences between non-sCSD and sCSD patients

Parameter	non-sCSD	sCSD	*p* value
Actual age (months)	139 ± 101	60 ± 70	< 0.05
Gestational age (weeks)	39 ± 2	37 ± 3	< 0.05
Birth weight (g)	3136 ± 554	2900 ± 448	n.s.
Plasma Na (mmol/L)	140 ± 2	120 ± 4	< 0.001
Plasma pH	7.34 ± 0.07	7.18 ± 0.11	< 0.001
Urinary Na (mmol/L)	139 ± 64	10 ± 4	< 0.001
FE_Na_ (%)	1.2 ± 0.7	0.1 ± 0.0	< 0.001
uNa/uCrea (mmol/mmol)	25.0 ± 11.1	0.9 ± 0.4	< 0.001
Fecal Na (mmol/L)	29 ± 9	106 ± 33.2	< 0.001
Fecal pH	6.5 ± 1.1	7.3 ± 1.1	n.s.

Abbreviations in alphabetical order: *FE*_*Na*_ fractional excretion of sodium; *mo* month; *n.s.* not significant; *uNa/uCrea* urinary Na/urinary creatinine ratio; *wk* week

## Discussion

It has been previously shown that mutations in *SPINT2* are associated with sCSD [7], a disease featuring a sodium-losing diarrhea, recurrent corneal erosions and frequently atretic malformations of the choanae (CA) and the gastrointestinal tract (GA). Furthermore, intestinal biopsies have shown tufts, consisting of focal crowds of enterocytes within the epithelium [16]. Mutational analysis of *SPINT2* had previously identified five distinct homozygous or compound heterozygous mutations [7] in each of the investigated sCSD patients (Table 1, lower panel). Our study was undertaken to investigate whether the *SPINT2* mutations identified in sCSD would also be present in patients with non-syndromic forms of isolated atresias, who did not have diarrhea (non-sCSD).

No homozygous or compound heterozygous *SPINT2* mutations were identified in isolated non-sCSD atresia subjects. However, in the non-sCSD patient #14 we found a heterozygous splice mutation, c.593-1G>A, which is identical to a *SPINT2* mutation shown previously in five Austrian sCSD patients [7]. This patient also had borderline laboratory values (Table 1) for sodium balance, indicating potential sodium depletion. This suggests that carriers of the c.593-1G>A *SPINT2* mutation might be recognizable by testing for phenotypical laboratory markers of sodium status (e.g., FENa, uNa/uCrea).

Regulation of the cell surface serine protease matriptase by the *SPINT2*-encoded hepatocyte growth factor inhibitor 2 (HAI2) has been shown to be essential for organogenesis [17]. Furthermore, loss of SPINT2 protein has been implicated in severe clefting of the embryonic ectoderm in mice [18]. This also raises the possibility of an influence of *SPINT2* on the development of CA and GA during prenatal organ formation. Another mechanism by which *SPINT2* mutations may lead to atretic malformations could be related to the function of the epithelial sodium channel (ENaC). This channel has been shown to be regulated by alterations of the serine protease–protease inhibitor balance which may, if defective, disturb the volume of the epithelial surface liquid layer [19]. Such a mechanism has been shown to be the case for the airway epithelium of cystic fibrosis (CF) patients in which hyperabsorption of sodium (and fluid) causes increased viscosity of the epithelial surface liquid. An analogous pathomechanism of epithelial fluid–electrolyte imbalance due to malfunction of the cystic fibrosis transmembrane regulator (CFTR) could be imagined to cause adhesion of epithelial layers inducing the commonly encountered aplasia of the vas deferens in CF [20].

## Conclusion

Our study aimed at investigating whether a similar mechanism of epithelial adhesion potentially related to *SPINT2* mutations could be responsible for the development of isolated CA and/or GA. However, our finding that no homozygous mutations were identified in the non-sCSD group argues against the possibility of *SPINT2* mutations being a cause of isolated CA or GA.
